# Evaluating the impact of highway construction projects on landscape ecological risks in high altitude plateaus

**DOI:** 10.1038/s41598-022-08788-8

**Published:** 2022-03-25

**Authors:** Chao Li, Jingxiao Zhang, Simon P. Philbin, Xu Yang, Zhanfeng Dong, Jingke Hong, Pablo Ballesteros-Pérez

**Affiliations:** 1 School of Economics and Management, Chang’an University, Xi’an, 710061 Shaanxi China; 2 School of Civil Engineering, University of Science and Technology Liaoning, Liaoning Anshan, 114051 China; 3 Nathu Puri Institute for Engineering and Enterprise, London South Bank University, London, UK; 4 Harbin Normal University, Harbin, 150500 Heilongjiang China; 5 Institute of Eco-Environmental Policy and Management, Chinese Academy of Environmental Planning, Beijing, 100012 China; 6 Chongqing University (CQU), Chongqing, 400044 China; 7 Departamento de Proyectos de Ingeniería, Universitat Politècnica de València, Camino de Vera s/n, 46022 Valencia, Spain

**Keywords:** Ecology, Environmental sciences

## Abstract

In China and other countries, many highway projects are built in extensive and high-altitude flat areas called plateaus. However, research on how the materialisation of these projects produce a series of ecological risks in the landscape is very limited. In this research, a landscape ecological risk analysis model for high-altitude plateaus is proposed. This model is based on the pattern of land uses of the surrounding area. Our study includes buffer analysis, spatial analysis, and geostatistical analysis. We apply the model to the Qumei to Gangba highway, a highway section located in the southeast city of Shigatse at the Chinese Tibet autonomous region. Through global and local spatial autocorrelation analysis, the spatial clustering distribution of ecological risks is also explored. Overall, our study reveals the spatial heterogeneity of ecological risks and how to better mitigate them. According to a comparison of the risk changes in two stages (before and after the highway construction), the impact of highway construction on the ecological environment can be comprehensively quantified. This research will be of interest to construction practitioners seeking to minimize the impact of highway construction projects on the ecological environment. It will also inform future empirical studies in the area of environmental engineering with potential affection to the landscape in high-altitude plateaus.

## Introduction

Since the beginning of the twenty-first century, the Chinese government has implemented specific construction plans for highways in the Tibet plateau. This has resulted in a major increase in the size and capacity of the highway network in this region^1^. However, the complex and special geological environment of the Tibet region has not been adequately considered in the construction process creating many negative effects. The progressive destruction of vegetation during highway construction has caused significant landscape changes along the highway route resulting in a fragmented landscape. At the same time, waste discharges and exhaust gases during the construction process have eroded the soil and will surely create further soil erosion and water pollution problems^2^. Recent research has been mainly focused on evaluating the impact of highway construction on urban areas^3–5^, lakes and river basins^6,7^ and nature reserves^8,9^. However, hardly any research has investigated the impact of highway construction in ecosystems at high altitudes in the Plateau. In the case of Tibet, the complex terrain, climatic conditions and the difficulty of data acquisition, has resulted in very limited research.

The Tibet plateau is a densely distributed area of nature reserves and an important ecological security barrier between China and the wider continent of Asia. It plays an important role in climate regulation, soil and water conservation, biodiversity protection and carbon accumulation. However, due to the fragility of the very cold environment and its sensitivity to external disturbances, the overall landscape pattern of such areas can be easily fragmented (i.e. acquire poor stability and resilience to external changes). Consequently, intensive highway construction will likely result on major negative impacts on the biodiversity and landscape patterns of this area.

At present, methods for ecological risk assessment include various modelling approaches from diverse fields like physics, chemistry and biology. Many of them involve simulation as well as other risk measurement methods, such as the expert judgment. Physics, chemistry and biological-based simulation involve developing a model in order to observe and test the envisaged impact of events on the ecological properties of the ecosystem, including biological life. The general risk measurement method is used to estimate the importance of the risk, which is broken down in probability and severity. When there are many system property uncertainties or just different opinions on the ecological value of some of the system components, the expert judgment method can also become an alternative method. Similarly, ecological risk assessment methods based on remote sensing technologies and geographic information systems are becoming increasingly common. These technologies are also used to process and analyze changes in the ecological environment^10,11^. In terms of ecological risk evaluation indicators, there tends to be two main aspects analyzed: heavy metal pollution in soils and landscape pattern changes. Landscape ecological risk assessment pays more attention to spatio-temporal heterogeneity. Understanding this heterogeneity can help decision-making for regional risk prevention and improve landscape management^12,13^. However, empirical studies that confirm the advantages and application of these models are extremely limited.

This research study builds on existing research and adopts the Qumei to Gangba highway section in the southeast city of Shigatse. This city is located in the Tibet autonomous region of China. The study considers the 10-km buffer zone along the highway in a high-altitude plateau area and develops a landscape ecological risk assessment model based on the landscape pattern. The spatial and temporal distribution of the landscape ecological risks along the highway are evaluated before and after the highway construction. This analysis reveals the characteristics and extensive impacts of highway construction on the landscape pattern and the landscape ecological risks of the area. It will also provide an improved understanding of the technical support required for the post-construction stage of ecological restoration in these high-altitude areas.

## Literature review

### The impact of infrastructure construction on landscape patterns

Engineering construction activities deeply affect the regional landscape and the level of biological activity, including human life. On the microscopic scale, construction activities frequently result in heavy metal pollution, which change the structure of the surface soil and affects the migration of species^14^. On the landscape scale, construction activities often lead to long-term fragmentation.

Based on GIS and the buffer analysis method, Minxi et al.^15^ used several landscape metrics to quantitatively study the impact of hydropower projects construction on landscape structure changes. Yang et al.^16^ investigated the process of hydropower facility development and the ecological characteristics of a river basin. Then, they systematically evaluated the impact of cascade hydropower development on the river landscape. Chen et al.^17^ adopted the West–East gas pipeline project in China as the research object, and studied its impact on the landscape pattern by comparing the changes of landscape indicators along its route.

In terms of research on the impact of highway construction on the landscape pattern, Qianqian et al.^18^ used remote sensing data to compare the pre- and post-construction stages in a 500-m buffer zone along the highway. Yong et al.^19^ analysed a 15-km buffer zone on both sides of the Yuyi expressway (Chongqing section). With the help of ArcGIS technology and landscape ecology methods, these researchers explored gradient differences of land use and landscape pattern evolution. Keken et al.^20^ monitored the land cover typologies in Czech Republic’s highways for nearly 60 years and studied the impact of road construction and operation on changes in landscape structure. Huang and Ting^21^ studied the impact of road construction on landscape fragmentation and evaluated their influence and environmental variables. Mothorpe et al.^22^ studied the impact of the American intercontinental highway construction on land use, and provided technical support for future agricultural land protection plans. Huang et al.^23^ investigated Taiwan’s township roads and showed that road construction led to varying degrees of isolation and fragmentation on the overall landscape pattern.

The above researches provide an effective method for in-depth evaluation of the changes of regional landscape pattern in the process of engineering construction, but it does not consider the vulnerability and particularity of plateau landscape under extremely complex geological conditions. Therefore, with the help of the above research methods, it is necessary to evaluate the evolution characteristics of landscape pattern in the process of highway construction in high-altitude Plateau, and the research results can provide differentiated governance schemes for landscape restoration in similar regions.

### The impact of infrastructure construction on ecological risks

With the rapid development of infrastructure development, the ecological impact of these works has caused higher stress levels in the surrounding environment. Many Chinese scholars have focused on theory and methods development of landscape ecological risks assessment. Consequently, a research framework of landscape ecological risk assessment has already been developed.

However, the landscape factors that have been considered are mainly focused on the quantification and analysis of landscape patterns. These research results have generally been used to characterize landscape ecological risks indirectly, though. In this vein, different scholars have selected relevant indicators, methods and models and applied them to different regions and for different evaluation purposes. For example, Jian et al.^24^ proposed environmental protection measures for tunnel construction by evaluating the ecological risks and provided a reference for the construction of similar geotechnical projects. Wang^25^ studied the ecological environment along a high-speed railway project and put forward several measures to prevent its disturbance. He and Xiong^26^ selected several indicators and analyzed their impact on the ecological environment with the aim of reducing ecological risks. Jing et al.^27^ verified the impact of the construction of a cross-sea bridge on the water quality and ecological environment of the surrounding sea. Jianhua et al.^28^ used multi-scale and multi-source remote sensing data to monitor and analyze the ecological environment and socio-economic impacts along a railway project. Fang^29^ analyzed the main ecological environmental problems and the main influencing factors of metal mine construction projects. Then, some methods for ecological environment assessment were proposed for those mine projects.

In terms of the impact of highway construction on ecological risks, many researchers have also adopted different study perspectives (from micro and macro frameworks, and from single roads to whole road networks). Bian et al.^30^ assessed the ecological and health risks of the surrounding areas of a highway project by analyzing the persistence of heavy metals in the soil. Limin et al.^31^ quantitatively studied the gradient changes of the landscape pattern in different buffer zones of roads according to an ad-hoc landscape pattern index. Liang and Nianlai^32^ adopted an index evaluation method to analyze the impact of highway construction on the surrounding ecological environment. Specifically, they focused on how to alleviate the impact of highway construction to maintain the sustainable use of local natural resources. Ting and Zongmin^33^ proposed an ecological environment impact assessment index system for highway construction. And more recently, Igondova et al.^34^ proposed another ecological impact assessment method for roads construction. In this case, though, their method included an ecological risk assessment index which could be applied in quantitative research studies.

### Research on landscape ecological risk assessment methods

Landscape ecological risk assessment frequently encompasses two methods: those based on risk sources, and those based on sink and landscape patterns. The so-called early landscape ecological risk assessment is one of the former methods, but it is not applicable when the regional ecological stress factors are not clear^35,36^. Evaluation methods based on landscape patterns directly evaluate landscape ecological risks from spatial patterns on a regional scale. In this regard, ecological risk assessment methods based on land use and cover changes have become a research hotspot^37^.

In the process of ecological risk assessment, the construction of landscape ecological risk indices based on landscape patterns or land mosaic patterns have become prominent too^38^. The risk level of the landscape can also be measured from the existence of external forces (i.e. rapid urbanization) and the internal pressure capacity of the landscape^39^. In terms of evaluation indicators, the expert scoring method is frequently used as research method. The sorting normalization method is also used as well, but the assignment method is conditioned by some subjective weight normalization which can affect the eventual assessment of different evaluation indicators^40,41^. As a systematic method, a multi criteria decision making (MCDM) method can reduce the interference of decision-makers' subjective judgments on decision-making, and is widely used in various fields of ecological risk assessment^42,43^. Among the MCDM methods, the analytic hierarchy process (AHP) method and the technique for order preferences by similarity to ideal solutions (TOPSIS) method are commonly used methods. Peng et al.^44^ used AHP to determine the weight of each factor affecting wetland ecological risk, and established a risk assessment model. Based on the improved AHP, Zhang et al.^45^ combined the fuzzy comprehensive evaluation (FCE) method and applied the improved AHP to the ecological environment impact assessment of expressways, and concluded that the improved method has good objectivity and reliability, applicability. Luan et al.^46^ used TOPSIS method to conduct multi criteria decision making analysis on environmental pollution caused by land cover change, and the research results provided scientific guidance for regional environmental management and planning. In addition, the combination of AHP and TOPSIS method has become an important method for ecological risk assessment^47,48^.

Recently, landscape ecological risk assessment methods usually accommodate multi-element ecological risk assessment. However, the selection of evaluation indicators remains subjective. Also, there is a lack of quantitative standards for ecological risks. Most of the evaluations are based on qualitative analysis, whereas quantitative analysis still is in an exploratory stage. Consequently, a comprehensive ecological risk assessment system needs to be established as soon as possible. Namely, a standard method for ecological risk assessment should be determined to provide a theoretical basis for further ecological environmental management and risk prevention measures. This method will be applied to highway construction projects, unlike many prior studies which have focused on cities or river basins.

## Research area and research methods

### General situation of the study area

The extensive repair work for the highway maintenance of the Qumei to Gangba highway section commenced in early 2019. This highway is located in the southeast city of Shigatse, in the Chinese Tibet autonomous region. The route is 145.64 km long and runs through three counties and seven towns. The terrain has a high altitude in the middle and low altitude at both ends. The highway runs along riverbed terraces and at the foot of mountain slopes. The average altitude of the highway is around 3850–4750 m above sea level.

The geomorphology of the area where most of the highway is located is a plateau valley landform with some mid-mountain landforms. Wide river valley areas are mostly U-shaped valleys and narrow V-shaped river valleys. The landforms are mainly developed as modern riverbeds, floodplains and river terraces accumulating and cutting out structural landforms. High terraces are generally developed in the valley area, with high mountains on both sides of the valley and steep slopes. The main source of replenishment for the river is rainfall water. Most of the highway track is located at the foot of the mountain slope where the topography is relatively even.

**Figure 1:**
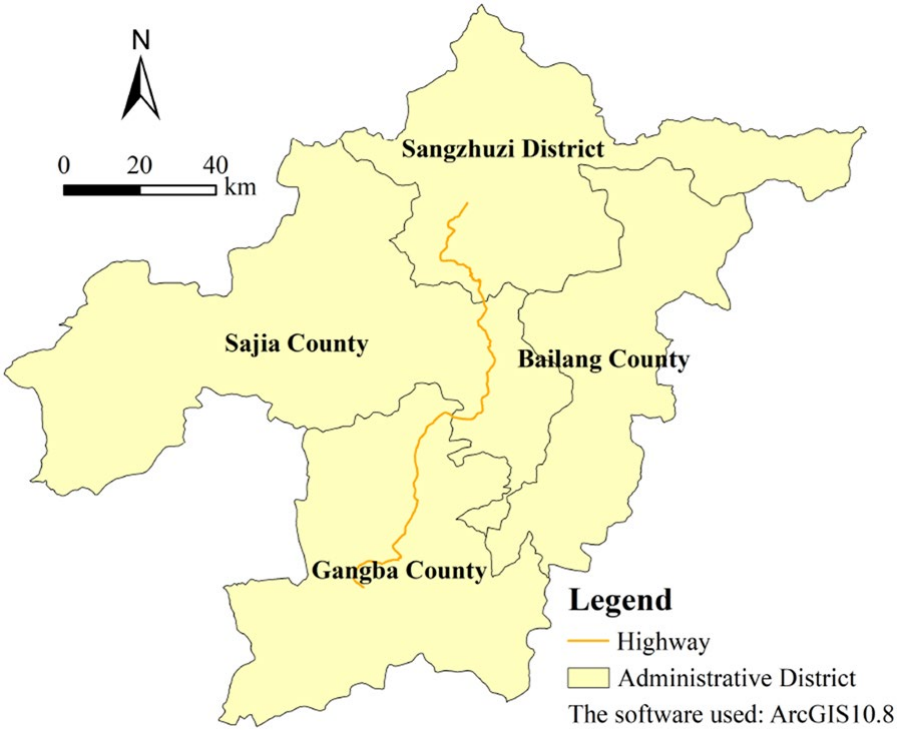
Location of the highway section from Qumei to Gamba.

The area where the route is located has a continental climate. The main features are dryness and lower levels of oxygen due to altitude, sufficient sunshine, and a wide temperature difference between day and night. Meteorological records from the Chinese Meteorological Bureau from recent years show that the annual average temperature of Sajia County is 5.5 °C, and the annual precipitation is 35 mm. The Sangzizhu area has a dry climate and thin air, with an average annual temperature of 4.9 °C and an average annual rainfall of 430 mm. The terrain of Gamba County is complex with large vertical changes. The highest point is 6783 m above sea level, whereas the lowest is 4375 m above sea level (a height difference of 2.408 m), and the annual average temperature is 1.5 °C. We used ArcGIS software to extract the vector boundary of some counties in Shigatse region, and superimposed the vector data of highway from Qumei to Gamba in the software. Finally, we got the location of the highway (Fig. 1).

### Model construction

#### Buffer analysis

**Figure 2:**
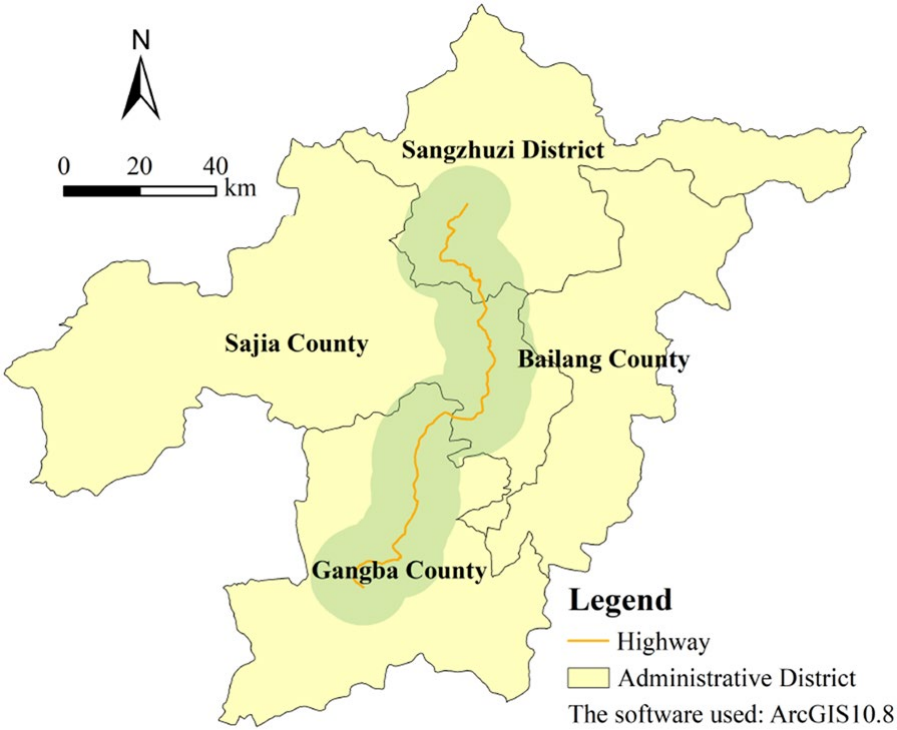
10-km buffer zone of the highway from Qumei to Gamba.

A highway is a linear structure, and its impact on the ecological environment is generally located on both sides of the highway. Buffer analysis is an analysis tool for studying the ecological and environmental effects of roads and other linear structures. Based on this method, the spatial variation of the impact of roads on a certain indicator can be studied by comparing the spatial differences of related indicators in buffer zones. This method has been widely used in the fields of ecology and pollution evaluation^49^. Like previous research, on the basis of Fig. 1, this study uses the highway as the baseline and uses the buffer analysis tool built in ArcGIS10.8 to generate a 10-km buffer zone on both sides. The influence process of highway construction on the landscape pattern and ecological risk is discussed indirectly by using the differences measured in each index and buffer zones (see Fig. 2).

#### Construction of the ecological risk index

Ecological risk refers to the probability of which a regional ecosystem can remain stable in response to external disturbances (including natural and/or human activities). A landscape ecological risk model is a method that quantifies ecological risks by considering internal and external factors of the ecosystem. Hence, an ecological risk index model evaluates the ecological risk status of a region from the landscape characteristics such as the landscape disturbance index that characterizes the degree of external disturbance. It also uses the landscape vulnerability index to describe the ability of the ecosystem to maintain stability.

The landscape ecological risk index along the highway is composed of two parts, namely the landscape disturbance index and landscape vulnerability index. The Landscape disturbance index (*E*_*i*_) includes three factors: the landscape fragmentation index (*C*_*i*_), the landscape separation index (*S*_*i*_) and the landscape dominance index (*Di*). These indices are weighted to interpret some system results.

1. Landscape disturbance index (*E*_*i*_)

The landscape disturbance index can reflect the disturbance degree of different landscape types. We select here a landscape fragmentation index, landscape separation index and landscape dominance index to build the Landscape disturbance index^50^.

- Landscape fragmentation index (*C*_*i*_)

Landscape fragmentation is a process in which the regional landscape structure gradually tends to be complex, heterogeneous and discontinuous under the action of external factors. The landscape fragmentation index has important ecological significance in measuring the loss of biodiversity. This index is calculated as:

1 $${C}_{i}={n}_{i}/{A}_{i}$$

In (1), *n*_*i*_ is the number of patches of landscape type *i* and *A*_*i*_ is the total area of landscape type *i*.

- Landscape separation index (*S*_*i*_)

The landscape separation index is used to describe the degree of separation between landscape patches and quantify the connection between various ecosystems. Its expression is:

2 $${S}_{i}=\sqrt{\frac{{n}_{i}}{A}}\times \frac{A}{2{A}_{i}}$$

In (2), *A* is the total area of the landscape.

- Landscape dominance index (*D*_*i*_)

The degree of landscape dominance is the difference in the areas of various patches. It describes the degree in which the overall landscape is dominated by the main landscape types. The greater the landscape dominance grows (or falls), the higher the increase (decrease) in area ratio differences of every landscape type. The calculation formula is:

3 $${D}_{i}=\frac{{R}_{i}+{F}_{i}}{4}+\frac{{L}_{i}}{2}$$

In (3), *R*_*i*_ is the ratio of the number of risk evaluation units with landscape type *i* respect to the total number of risk evaluation units; *F*_*i*_ is the ratio of the number of patches with landscape type *i* to the total number of patches in the evaluation unit; *L*_*i*_ is the ratio of the area of landscape type *i* to the total area of the evaluation unit.

After calculating the three indices of *C*_*i*_*, S*_*i*_ and *D*_*i*_ according to formulae (1)–(3), a normalization has to be performed. This research assigns the following weights for the fragmentation, separation and dominance indices: 0.5, 0.3, and 0.2, respectively. The calculation formula of the landscape disturbance index for each land cover type of each risk assessment unit is obtained as follows:

4 $${E}_{i}=0.5{C}_{i}+0.3{S}_{i}+0.2{D}_{i}$$

(2) Landscape vulnerability index (*F*_*i*_)

The landscape vulnerability index refers to the ability of the regional ecosystem to resist external disturbances. This is the internal factor that characterizes ecological risks. This index is closely related to the stage of the landscape in the process of natural alternation. According to classification of land uses, the landscape of the study is divided into six main types: cultivated land, wood land, grass land, water land, construction land, and unused land. Also, based on previous studies and the actual conditions of the Tibet region (Yanxu et al. 2015; Qiran and Hui 2014), the expert scoring method is used to determine the value of the landscape vulnerability along the highway. The value of landscape vulnerability index for each land type is deemed as follows: unused land is 6, water land is 5, cultivated land is 4, grass land is 3, wood land is 2, construction land is 1, and then normalized to obtain various types of landscape vulnerability indexes. The values above then become 0.2857, 0.2381, 0.1905, 0.1429, 0.0952, and 0.0476, respectively.

(3) Landscape ecology risk index (*ERI*_*k*_)

In order to connect the internal composition of the landscape pattern with the regional ecological risk status, and combine it with the above indices, this study uses the area proportions of each landscape type. This way, a landscape ecological risk index model is built. This index model can be used to describe the relative ecological properties loss of a certain sample area. The latter fully reflect the changes in ecological risks caused by the change of landscape pattern. The specific construction of the model is as follows:

5 $${ERI}_{K}=\sum_{i=1}^{n}\frac{{A}_{i}}{A}\left({E}_{i}\times {F}_{i}\right)$$

In (5), *ERI*_*k*_ is the ecological risk index value of the kth risk assessment unit; *A*_*i*_ is the area of land cover type *i* in the kth risk assessment unit; *A* is the area of the kth risk assessment unit; *E*_*i*_ and *F*_*i*_ are the value of landscape disturbance index and landscape vulnerability index of land cover type *i* in the kth risk assessment unit, respectively.

#### Spatialization of ecological risks

1. Division of the evaluation unit

According to the scope of the study area and the sampling workload, we proposed a method for combining point grid evaluation units with the area vector evaluation units. If the grid is too large, it cannot reflect the spatial difference. If the size is too small, the landscape type is too single and the calculation is too large. Referring to previous studies, the grid should be 2–5 times of the average patch area^51,52^. Therefore, on the basis of considering the actual situation and workload of the research area, a square of 2 km × 2 km was used as the smallest area (unit) to calculate the landscape comprehensive index. The sampling method was equidistant for all squares. This process can be easily performed with ArcGIS. This way, 820 different risk units were analyzed, and the comprehensive ecological risk index was calculated for each of them. Then, the ecological risk level at the center point of each 2 × 2 km^2^ sample area was used as the representative location of the index evaluation.

(2) Spatial autocorrelation analysis

Global spatial autocorrelation is a description of the spatial characteristics interrelation of several attribute values of an entire region^53^. It is used to test whether the value of a spatial variable is related to the value of the same variable in the adjacent space. This study uses Moran's I coefficient to reflect the similarity of the attribute values of spatially adjacent regional units. The formula for calculating the global Moran’s I coefficient is:

6 $$I=\frac{\sum_{i=1}^{n}\sum_{i=1}^{n}{W}_{ij}\times \left({X}_{i}-\overline{X}\right)\left({X}_{j}-\overline{X}\right)}{\sum_{i=1}^{n}\sum_{i=1}^{n}{W}_{ij}\times \frac{1}{n}\sum_{i=1}^{n}{\left({X}_{i}-\overline{X}\right)}^{2}}$$

In (6), *W*_*ij*_ represents the spatial connection matrix between spatial unit *i* and *j* with *i* ≠ *j*; *n* is the total number of spatial units; *X*_*i*_ is the attribute value of the spatial unit *i*; *X*_*j*_ is the attribute value of spatial unit *j*; and $\overline{X}$ is the attribute average value of all spatial units. The value of *I* ranges from − 1 to 1. When *I* = 0, it means that the space autocorrelation is irrelevant. When *I* takes a positive (negative) value, there is a positive (negative) correlation.

Local spatial autocorrelation analysis can also be applied when: it is necessary to consider if there is a local spatial aggregation of high or low values of observations; when we want to find out which regional unit contributes more to the global spatial autocorrelation; and when we want to find to what extent the global assessment of the spatial autocorrelation conceals some abnormal local conditions. This study used the local autocorrelation LISA index to analyze the spatial aggregation of high and low values of the regional ecological risk index. Then, we explored the abnormal areas of the ecological risk distribution by identifying potential high or low “hot spots” with significant ecological risks. The formula for the LISA index is as follows:

7 $$\mathrm{LISA}=\frac{\left({X}_{i}-\overline{X}\right)}{\sum_{i}{\left({X}_{i}-\overline{X}\right)}^{2}/n}\sum_{i}{W}_{ij}\left({X}_{j}-\overline{X}\right)$$

### Data source and processing

The basic data used in this study mostly encompassed remote sensing image data, vector data of the highway and administrative area, as well as land use and land cover data.

1. Remote sensing image data. The large and medium-level repair of the highway maintenance works on the Qumei to Gamba section of the highway took place between early 2019 and October 2020. In addition, because the characteristics of ground classes in high altitude areas of the plateau are often not obvious, the accuracy of selected training samples may not be guaranteed if low-resolution images are used, which may reduce the accuracy of classification. This study selected Sentinel-2 remote sensing images taken in October 2016, October 2018, and October 2020. The data was acquired from the European Space Agency’s (ESA) website with a resolution of 10 m. This raw source data went through some image processing such as orthorectification, registration, and band fusion. The preprocessed data as a data source for supervised classification (Table 1).
2. The vector data of the highway and administrative area. The administrative area data was mainly gathered from the administrative boundary data of the Shigatse area, including the administrative boundaries and administrative centers of the township-level regions. The vector data of the highway from Qumei to Gamba section of the highway was also collected. This data was sourced from the Resource and Environmental Science and Data Center of the Chinese Academy of Sciences (https://www.resdc.cn/).
3. Land use and land cover data. With the support of ENVI5.3 software and ArcGIS10.8, a support vector machine algorithm was used to supervise and classify the preprocessed remote sensing images. The sentinel-2 image data of different phases were also automatically interpreted and the accuracy of the data remained always above 90% (Table 1). Finally, we took cultivated land, forest land, grassland, water area, construction land and unused land as the main landscape types in this study.

**Table 1:**
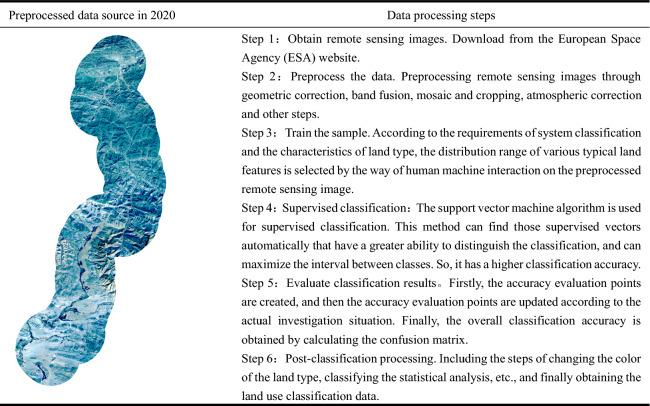
Data sources and data processing steps.

## Analysis and results

### Analysis of landscape pattern changes

**Figure 3:**
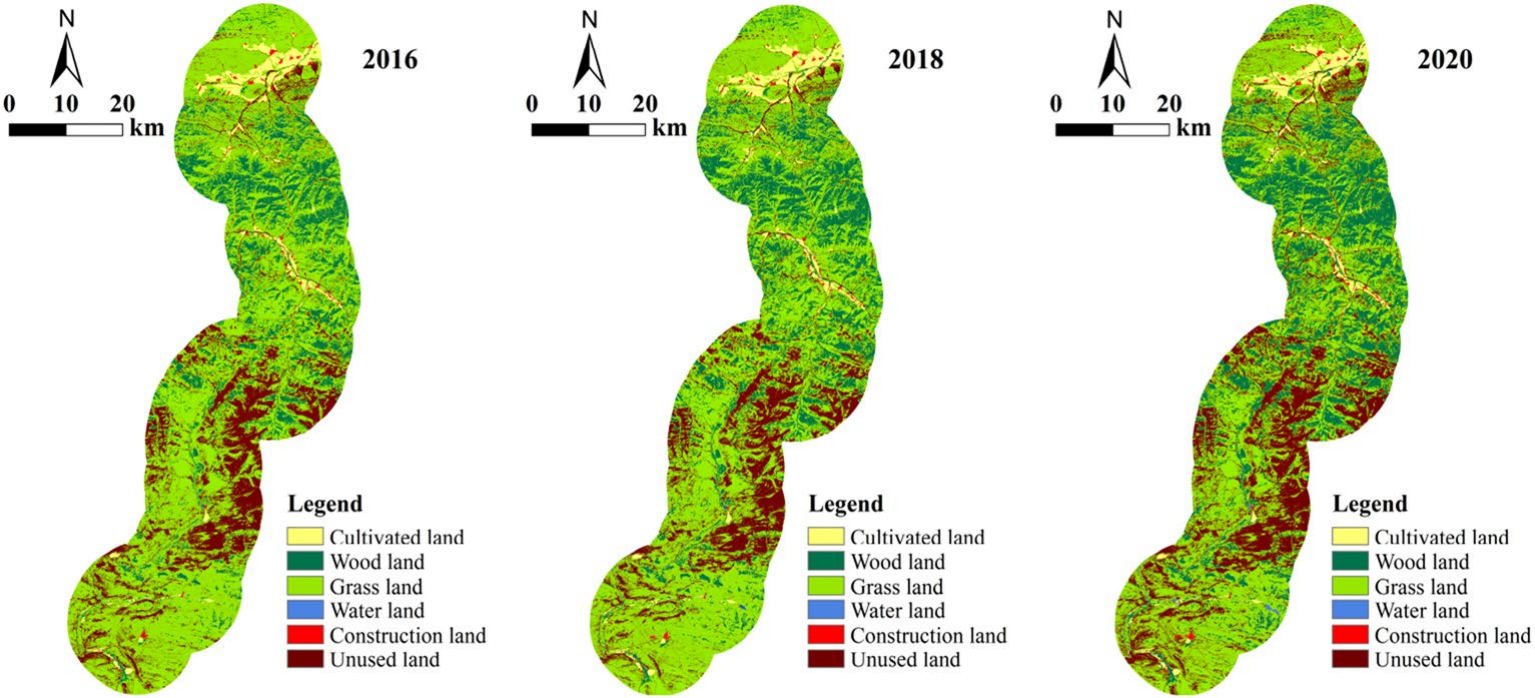
Map of land usages in the study area from 2016 to 2020.

According to the land use of the study area and the six-type land classification of the Chinese Academy of Sciences (cultivated land, wood land, grass land, etc.), the overall landscape pattern of the study area was analyzed. It can be observed in Fig. 3 that grass land, wood land and unused land were the main types of land use. This is common because of the topography of the Tibetan plateau region. Construction land was mainly distributed in several towns and villages such as the Qumei township, and along the highway. Cultivated land was distributed in large extensions around residential areas. The area of water land was the smallest, mainly distributed on both sides of the highway.

In order to fully show the influence law of highway construction on the landscape pattern, therefore, we estimated the land use transfer matrix along the highway in 2016–2018 and 2018–2020. By comparing the land use type transfer before and after highway construction, the influence of highway construction on landscape pattern was quantitatively evaluated. The calculation results are shown in Tables 2 and 3 respectively.

**Table 2:** 2016–2018 Land use area transfer matrix (km^2^).

Land type	Cultivated land	Wood land	Grass land	Water land	Construction land	Unused land	Total	Transfer out/%
Cultivated land	86.80	0	0	0.09	0.03	0	86.92	0.14
Wood land	0.12	417.64	0	0	0	0	417.76	0.03
Grass land	3.01	105.48	1666.17	0.57	0.68	67.62	1843.52	9.62
Water land	0.02	0	0.05	5.62	0	0.02	5.72	1.58
Construction land	0	0	0	0	14.62	0	14.62	0
Unused land	0.03	0.15	98.17	0.25	0.15	420.58	519.33	19.01
Total	89.98	523.27	1764.39	6.52	15.48	488.22	2887.87	–
Transfer in/%	3.53	20.19	5.57	13.80	5.56	13.85	–	–

**Table 3:** 2018–2020 Land use area transfer matrix (km^2^).

Land type	Cultivated land	Wood land	Grass land	Water land	Construction land	Unused land	Total	Transfer out/%
Cultivated land	87.25	0.32	1.39	0.09	0.74	0.18	89.98	3.03
Wood land	0.26	475.42	46.60	0.42	0.13	0.43	523.27	9.14
Grass land	3.71	140.45	1410.13	2.88	3.71	203.50	1764.39	20.08
Water land	0.17	0.23	0.65	4.94	0.02	0.52	6.52	24.23
Construction land	0.53	0.06	0.84	0.03	13.46	0.56	15.48	13.05
Unused land	0.17	1.24	49.26	1.82	1.36	434.37	488.22	11.03
Total	92.09	617.73	1508.88	10.18	19.42	639.57	2887.87	–
Transfer in/%	5.26	23.04	6.54	51.47	30.69	32.08	–	–

In the process of land type transfer, the main types of land type transfer are unused land to grassland (98.17 km^2^) and grassland to forest (105.48 km^2^). Unused land and grassland were turned out, accounting for 19.01% and 9.62% of their respective areas in 2016, respectively. Meanwhile, woodland, unused land and water area accounted for 20.19%, 13.85% and 13.80% of their respective areas in 2018, respectively. However, due to the small total area of water area, the converted area was less than 1 km^2^. It can be concluded from the transfer of land use types from 2016 to 2018 that the area of unused land was reduced before road construction, and the vegetation coverage was increased overall. Essentially, the overall transfer area of land use types was very small, and this transfer scenario was relatively simple.

It can be seen from Table 3 that the transfer of land use types is significantly more complicated after highway construction. The bidirectional transfer between grassland and unused land and between grassland and forest land is the main type of transfer. Among them, 203.50 km^2^ of unused land was transferred from grassland, followed by 140.45 km^2^ of unused land from grassland to woodland. Meanwhile, unused land and forest land were the main sources of grassland transfer, with 49.26 km^2^ and 46.60 km^2^ respectively, but their area was much smaller than that of grassland transfer.

Compared with the land use transfer before highway construction, it can also be seen that during highway construction, the transfer proportion of all land types increased significantly. The proportion of grassland and water area transferred out is more than 20%, and the area of water area, unused land and construction land transferred out account for 51.47%, 32.08% and 30.69% of the total area respectively.

According to the above analysis, it can be inferred that in the process of highway construction, grassland was damaged and polluted, and a large part of grassland was transferred to unused land, and the change of landscape pattern was more obvious than before highway construction, and the change of landscape pattern was more obvious after highway construction.

**Figure 4:**
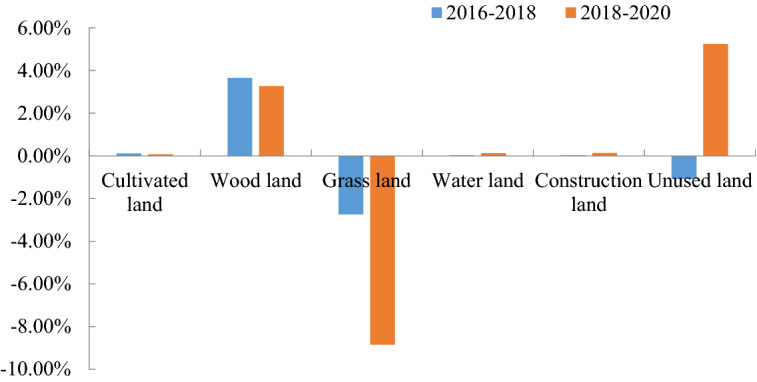
Changes in various landscapes (land use types) along the highway in 2016–2018 and 2018–2020.

Due to the differences in the distribution of land use types, the landscape also had a significant change over time. As can be deduced, the major changes involved the grass land, unused land and wood land (Fig. 4). Initially, grassland was the most abundant landscape type, and it accounted for more than half of the total area of study. Wood land increased by 3.65% in 2016–2018, and by 3.27% in 2018–2020. Conversely, grass land decreased by 2.74% and 8.85% across the two periods analyzed. The unused land showed opposite trends in both periods (first a decrease of 1.08% before the highway construction, then an increase of 5.24% after the highway construction).

In order to study the changes of landscape patterns quantitatively, we calculated the landscape ecological risk index with Fragstats4.2 software (see Table 4). The landscape indices of different landscape (land use) types also showed significant changes. Before and after the highway was constructed, the ecological risk index of cultivated land and construction land was the smallest. The reason was that the distribution of cultivated land and construction land was relatively concentrated, so the integrity of the patch improved. Unused land was highly fragmented and had the highest risk index, which was mainly related to the nature of land use in the landscape type. Combining the landscape indices of various landscapes, the ecological risk index along the highway in 2016, 2018, and 2020 was also calculated (with Formula 5). After its calculation, the ecological risk index of the three phases were 0.2316, 0.2217, 0.2822, respectively. Judging from the overall changes in the four years of the time span, the ecological risk index of the buffer zone did not change significantly before the highway was constructed. However, afterwards, the ecological risk index increased much more than in the previous period (a differential increment of 0.0605 and total increase of 27.29%). This variation indicated that the ecological risk index along the route showed a steeper upward trend after the highway was constructed.

**Table 4:** The landscape pattern index of different land types along the highway from 2016 to 2020.

Landscape type	Year	Landscape fragmentation index	Landscape separation index	Landscape dominance index	Landscape disturbance index	Landscape vulnerability index	Ecological Risk Index
Cultivated land	2016	0.0163	0.3684	0.0557	0.0009	4	0.0002
	2018	0.0222	0.4223	0.0590	0.0015	4	0.0003
	2020	0.0252	0.4443	0.0607	0.0020	4	0.0004
Wood land	2016	1.1567	1.4138	0.3404	0.3903	2	0.0372
	2018	1.0498	1.2037	0.3747	0.3630	2	0.0346
	2020	0.8537	0.9976	0.3923	0.4053	2	0.0386
Grass land	2016	0.5499	0.4640	0.6551	0.3060	3	0.0437
	2018	0.5435	0.4716	0.6379	0.2987	3	0.0427
	2020	0.6773	0.5695	0.5971	0.3728	3	0.0533
Water land	2016	0.1259	3.9868	0.0632	0.3233	5	0.0770
	2018	0.1303	3.7961	0.0634	0.3223	5	0.0767
	2020	0.0767	2.3329	0.0650	0.3155	5	0.0751
Construction land	2016	0.0116	0.7583	0.0684	0.0366	1	0.0017
	2018	0.0136	0.7959	0.0695	0.0368	1	0.0018
	2020	0.0165	0.7830	0.0715	0.0578	1	0.0028
Unused land	2016	2.7558	1.9576	0.4601	0.7667	6	0.2190
	2018	2.8222	2.0429	0.4520	0.7799	6	0.2228
	2020	2.1771	1.5682	0.4770	0.8337	6	0.2382

### Analysis of the temporal and spatial changes of the ecological risk index

#### Distribution characteristics of ecological risk index

**Figure 5:**
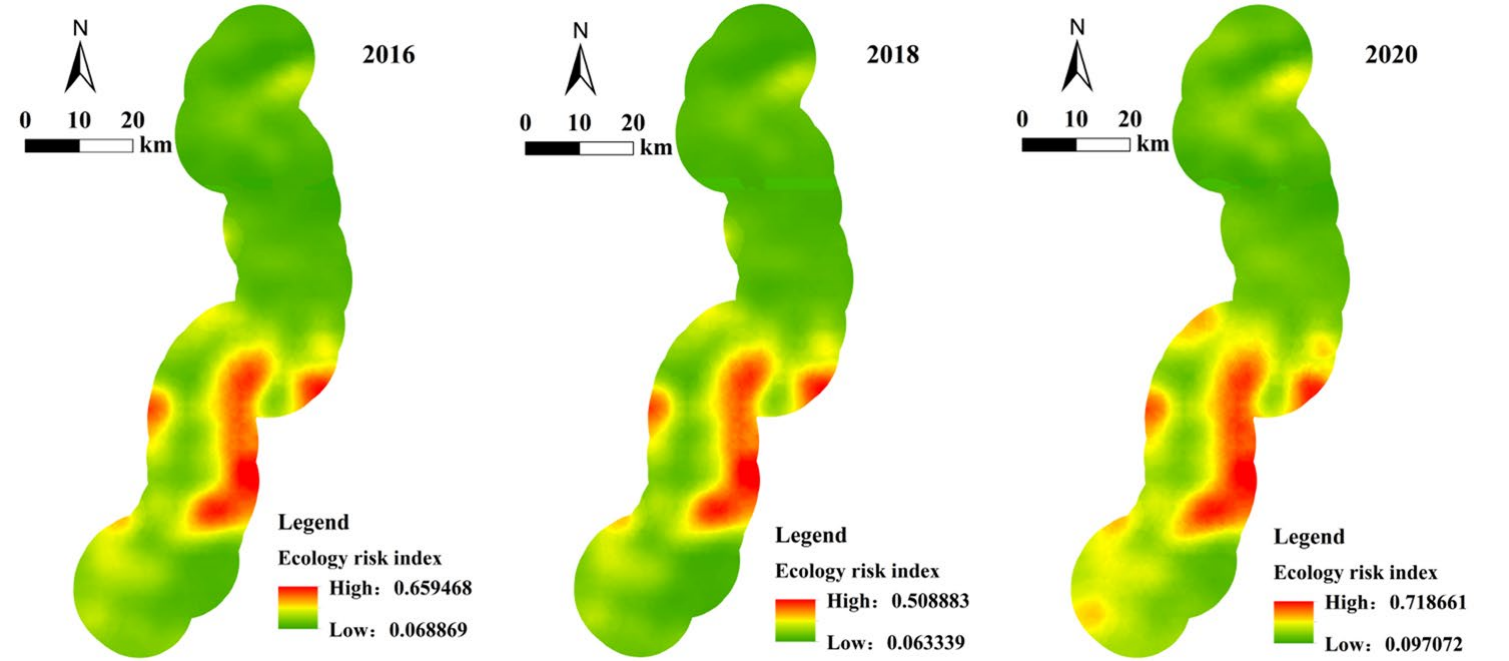
The distribution of ecological risk index along the highway in 2016, 2018, and 2020.

Based on the grid model and the Kriging interpolation method performed with the geostatistical analyst module of ArcGIS software, the ecological risk index of each risk assessment unit was spatially interpolated. This allowed us to create the ecological risk index spatial distribution map along the highway (see Fig. 5).

**Figure 6:**
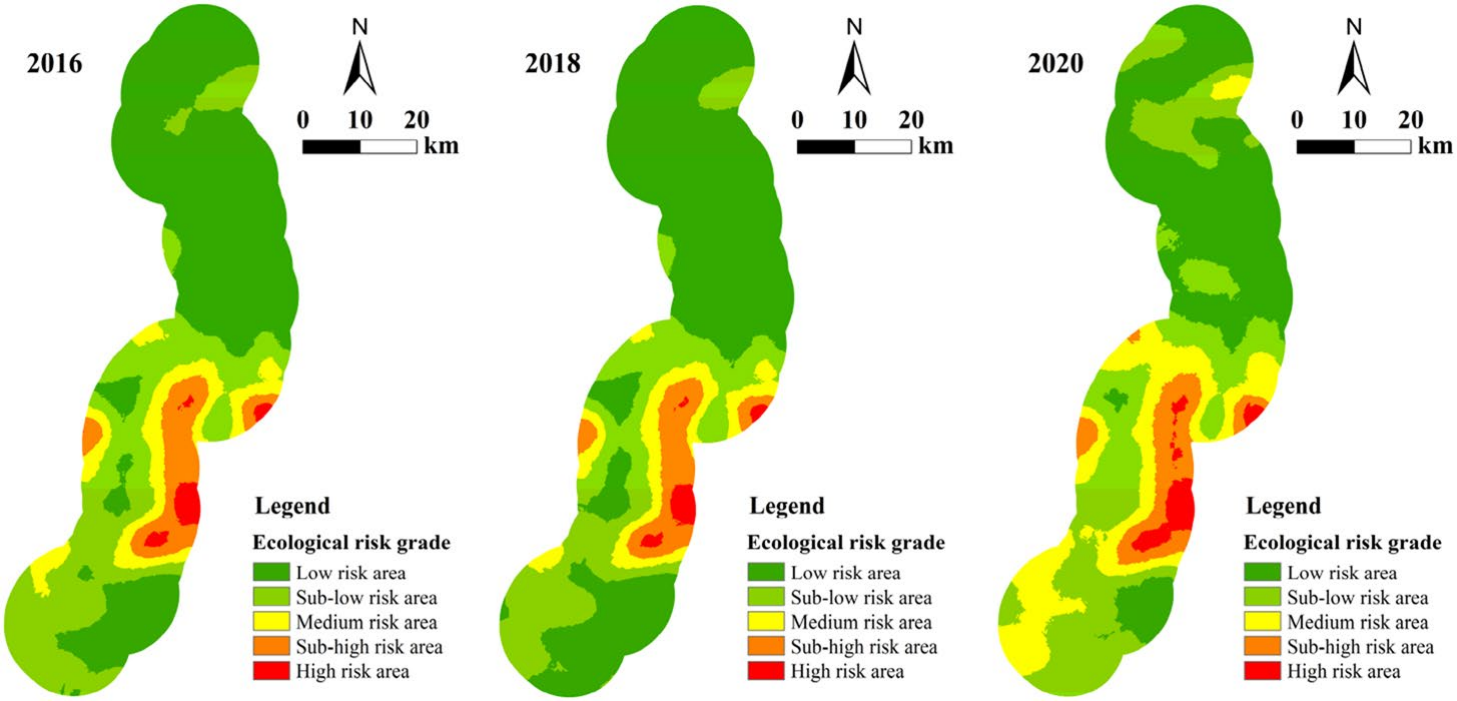
Distribution of ecological risk levels in 2016, 2018, and 2020.

In order to better discriminate the ecological risk changes in the areas along the highway and further study the impact of project construction, we created five ecological risk index value bins, namely: low risk area (0 ≤ ERI < 0.2), sub-low risk area 0.2 ≤ ERI < 0.4), medium risk area (0.4 ≤ ERI < 0.6), sub-high risk area (0.6 ≤ ERI < 0.8) and high risk area (0.8 ≤ ERI ≤ 1.0). An ecological risk level distribution map was drafted (see Fig. 6). It can be seen therefore that during the construction of the highway, the ecological risk level changed significantly. This was mainly manifested as a generalized shift from a lower level of risk to a higher level of risk in most areas.

#### Time series changes of the ecological risk index

In Table 5, the analysis highlights that the proportion of area occupied by the low risk area was the largest in the three years of the study (51.35%, 57.71%, and 36.65%, respectively). The area of low risk increased slightly before highway construction, then decreased sharply during the construction period (a decrease of 21.07%). The area change of the medium risk area was in second place (it increased by 10.58% during the construction of the highway). The area of high risk had the smallest proportion, but the maximum area appears in 2020, when reached the 2.76% of the total area. The sub-high risk area had the smallest amount of change in the three phases (area changes remained below 1% in the two periods). In general, before the road was constructed, areas with low ecological risk increased, while areas with high ecological risk slightly decreased. However, after the highway was constructed, the opposite change occurred. This indicated that the ecological security status during the construction of the highway affected the surrounding area to some extent.

**Table 5:** Changes in the ecological risk level areas along the highway from 2016 to 2020.

Ecological risk level	2016		2018		2020	
	Area/km^2^	Proportion/%	Area/km^2^	Proportion/%	Area/km^2^	Proportion/%
Low risk area	1482.93	51.35	1666.49	57.71	1058.42	36.65
Sub-low risk area	879.62	30.46	745.06	25.80	991.66	34.34
Medium risk area	269.80	9.34	233.66	8.09	539.30	18.67
Sub-high risk area	208.14	7.21	200.44	6.94	218.85	7.58
High risk area	47.37	1.64	42.21	1.46	79.64	2.76

Based on the previous classification of ecological risk grades, the analysis of ecological risk grade transfer changes helps to identify further local differences. Hence, with the help of a spatial statistical analysis method, the ecological risk transfer matrix of the 10-km buffer zone in two periods could be calculated (see Tables 6, 7).

**Table 6:** Transfer matrix of ecological risk levels in the 10-km buffer zone along the highway in 2016–2018.

Ecological risk level		Areas of different ecological risk levels in 2019/km^2^				
		Low risk area	Sub-low risk area	Medium risk area	Sub-high risk area	High risk area
Areas of different ecological risk levels in 2016/km^2^	Low risk area	1482.67	0.26	0	0	0
	Sub-low risk area	183.83	695.79	0	0	0
	Medium risk area	0	49.00	220.80	0	0
	Sub- high risk area	0	0	12.86	195.28	0
	High risk area	0	0	0	5.16	42.21

**Table 7:** Transfer matrix of ecological risk levels in the 10-km buffer zone along the highway in 2018–2020.

Ecological risk level		Areas of different ecological risk levels in 2020/km^2^				
		Low risk area	Sub-low risk area	Medium risk area	Sub-high risk area	High risk area
Areas of different ecological risk levels in 2018/km^2^	Low risk area	1049.07	613.89	3.53	0	0
	Sub-low risk area	9.35	377.76	357.54	0.41	0
	Medium risk area	0	0	175.97	57.69	0
	Sub-high risk area	0	0	2.27	159.90	38.28
	High risk area	0	0	0	0.85	41.36

In Table 6 we can see that the changes in the ecological risk exhibited the characteristics of a higher level of risk shifting to adjacent lower risks from 2016 to 2018. The total area of the reduced ecological risk level area was 250.85 km^2^, while the area with increased ecological risk level was just 0.26 km^2^. This indicates that the ecological security situation along the highway improved during this period. In the process of ecological risk transfer and transformation encompassing all levels, the dominant area transfer occurred from sub-low risk to low risk, with an area of 183.83 km^2^ accounting for 73.21% of the total transferred area. Overall, it can be observed that the ecological environment along the highway was generally better before the road was constructed.

In Table 7 we can see that the changes in ecological risk grade between 2018 and 2020 were still dominated by the transfer of adjacent risk levels. In this time span, the area of transfer across risk levels was minimal. During the period of highway construction, the ecological risk changes were mainly based on the transfer of low-level risks to adjacent high-risks. The total area of the areas with elevated risk levels was 1071.34 km^2^, which accounted for 37.10% of the total area. The area with reduced risk levels was 12.47 km^2^, which was far less than the total area with increased risk. These results highlight that the overall ecological risk along the road rose during the construction of the highway and the overall ecological security situation showed a downward trend.

### Spatial autocorrelation analysis of the ecological risk index

#### Global spatial autocorrelation analysis

With the aid of the “Geoda” software, we calculated the global Moran’s I index value of the ecological risk index in 820 2 × 2 km^2^ sample areas along the highway in 2016, 2018 and 2020. Namely, this calculation was used to verify the spatial pattern and significance of the ecological risk index of the entire study area.

The Moran’s I index in 2016, 2018 and 2020 was 0.954, 0.952 and 0.955, respectively. The index in the three phases was also positive, and its change trend not very obvious. However, this indicates that the ecological risk index of the area along the highway had a moderately strong positive correlation with the spatial distribution (i.e., adjacent plots had mutual influence and showed a high degree of spatial similarity). In the time series, the spatial clustering of plots with similar land use showed first a trend of ecological index decrease, and then, an increase. This indicates that the overall spatial differentiation of the landscape ecological risk intensity increased slightly along the route during the construction of the highway.

#### Local spatial autocorrelation analysis

**Figure 7:**
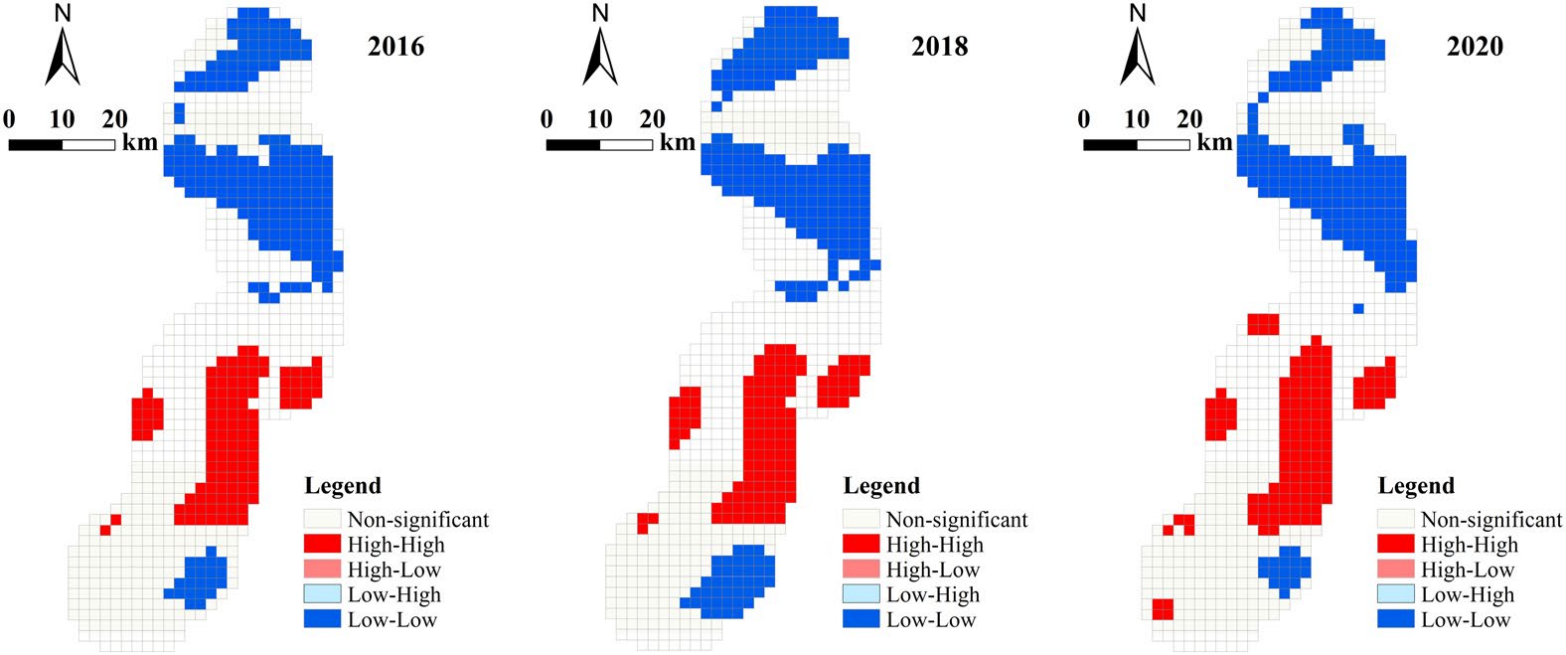
Local spatial autocorrelation diagrams of the ecological risk index.

The global autocorrelation analysis mainly considers the overall distribution of the ecological risk index. However, the local spatial autocorrelation helps to discriminate more fine-grained (local) change characteristics and spatial patterns. The LISA graph of local autocorrelation was used in this case to analyze the ecological index risk plots around the highway. Rook's adjacency weight matrix was also used to calculate the local autocorrelation results along the highway from 2016 to 2020 (see Fig. 7).

It can be observed in Fig. 7 that the types of the spatial distribution of the ecological risk index in the three phases was mainly of high–high aggregation and low–low aggregation. The low–low aggregation areas of ecological risk were mainly distributed in the northern and southeastern parts of the study area. This indicates that the ecological risk intensity in this region was low, and the ecological risk intensity of adjacent areas was also low. The latter may have happened because the area had more wood land and this ecosystem was more stable. The high–high aggregation areas of ecological risk were mainly concentrated in the central and southern part of the study area. This highlights that the ecological risk grade of these areas was high, and the ecological risk grade of adjacent areas was also high. This probably happened because the area had more unused land and low vegetation coverage. Finally, it can also be seen that the number of high–high concentration plots increased significantly from 2018 to 2020. This may have happened because grass land was extensively destroyed during the highway construction process, which further increased the area of unused land.

## Discussion

At present, studies on the impact of engineering construction on ecological risk are mainly focused on large-scale hydropower projects and urban roads in developed areas, while there is still a lack of systematic research on ecological risk assessment in high-altitude Plateaus. The process which highways impact the ecological environment is quite complex. This study has adopted the macro perspective of the landscape pattern and enabled the development of an ecological risk model. According to a comparison of the risk changes in two stages (before and after the highway construction), the impact of highway construction on the ecological environment has been quantified. Although there are certain limitations in this study, the analysis results are consistent with the actual survey situation for the highway.

Regarding extant research on the impact of highway construction on the ecological environment, most researchers have focused on time-based studies over an extended time period^20,54^. The research reported here adopted the time period corresponding to the highway works maintenance program. Remote sensing data analyzed in the study were selected from the same month of each year with a unified time scale. Furthermore, the landscape pattern and ecological risk indicators before and after highway construction were compared to study the impact of highway construction on the ecological environment. At the same time, due to the complex terrain in Tibet and the abrupt land features, in order to improve the classification accuracy, Sentinel-2 remote sensing images with a resolution of 10 m were selected. These images were combined with actual field surveys and, in some cases, incoherent data were corrected.

The analysis of landscape pattern before and after highway construction identified that the landscape pattern changes were more pronounced during the construction period than before construction. However, the overall landscape pattern as well as the types of main land uses along the highway did not change. This is in line with the conclusions of Gang, HuiJun, and Guang^55^ on wetlands in arid areas, and Haihang et al.’s^56^ on urban areas. The analysis of the ecological risk index before the highway construction scenario highlights that the average ecological risk index of the constructed highway is significantly higher than before. Although this is similar to the research study of Shiliang et al.^57^, it is fundamentally different to the conclusions of other researchers like Mo et al.^52^. A likely cause of this difference may be that the impact of construction on ecological risks of urban roads is different at the county-level and township roads, that is, urban areas may have more diversified risk sources.

For the convenience of discussion, we compared the differences between the relevant references and this study in methods, locations and main results in the form of tables (Table 8). It can be seen that some scholars have analyzed the ecological risk changes of river basins by establishing ecological risk models. The results show that in the process of urbanization, the ecological environment quality of these areas is declining, which is similar to the research conclusion of this paper^58,59^. Yuan et al.^58^ also analyzed the relationship between flood feature values and landscape patterns through multiple linear regression methods, which provided useful information for regional landscape planning and watershed flood control planning. Different from the above references, this study quantitatively studies the changes of various landscape indicators during highway construction, which can provide specific reference for regional landscape governance. At the same time, Research on the impact of infrastructure construction on ecological risk levels identifies that the transfer of ecological risks mostly involved the transfer between adjacent risk grades (i.e., cross-risk transfer rarely occurred). This is similar to the conclusions of Jie et al.^40^ on the ecological risks of the entire Qinghai-Tibet Plateau landscape. Furthermore, the research on the spatial heterogeneity identified that the ecological risk index of the three phases along the highway had a high degree of positive correlation with the spatial distribution. This spatial distribution was mainly of high-high aggregation and low-low aggregation. Particularly, the area with high-high aggregation increased significantly during the construction period. This is similar to the spatial distribution characteristics of the ecological risk index obtained by Fengjiao and Xiao^60^ and Xie et al.^59^. However, their research objects and methods were quite different.

**Table 8:** Comparison of research methods and main results with existing studies.

Reference	Study area	A	B	C	D	E	F	G	H	I	J	K	L	M
Xie et al.^59^	Jiangxi, China	11–40	100	×	√	×	√	√	√	×	×	×	/	↑
Oliveira et al.^62^	Minas Gerais, Brazil	100–1500	30	√	×	√	×	×	×	×	√	×	Wood land (Farmland)	/
Mo et al.^52^	Beijing, China	20–2303	30	×	√	√	√	×	√	×	×	√	Construction land (Cultivated Land)	↓
Dadashpoor et al.^64^	Tabriz, Iran	1300–3300	30	×	×	√	×	×	×	√	√	×	Construction land (Farmland)	/
Yuan et al.^58^	Nanjing, China	20–30	30	×	√	×	√	×	×	√	×	×	/	↑
Mann et al.^61^	Uttarakhand, India	204–2400	15	×	√	√	√	×	×	√	√	√	Construction land (Wood land)	↑
Li et al.^63^	Coastal Areas, China	4–10	260	×	×	√	×	×	×	√	√	×	Construction land (Cultivated Land)	/
This study	Shigatse, China	4375–6783	10	√	√	√	√	√	√	×	×	√	Unused land (Grass land)	↑

A: The main altitude of the study area, B: Remote sensing image resolution adopted (meter), C: Buffer analysis, D: Ecological risk model, E: Analysis of landscape pattern change, F: Analysis of ecological risk change, G: Global spatial autocorrelation analysis, H: Local spatial autocorrelation analysis, I: Regression analysis, J: Considerations of social or economic factors, K: Considerations of the impact of road construction, L: Landscape types with the largest area increase (decrease), M: Change of overall ecological risk (“↑” indicates increased risk, “↓” indicates decreased risk).

Relevant references also reported changes in regional landscape patterns and ecological risks caused by highway construction, which is similar to the method used in this study^52,61^. By analyzing the changes in the landscape pattern, these two references concluded that the landscape type with the largest area increase was construction land, while the area of unused land increased the most in the study. This may be caused by the large differences in population, economy, topography and other factors in the study area, which is located at extremely high altitude. It is worth mentioning that the highway is a single linear project in this study, so the research method of buffer zone is also adopted. Oliveira et al.^62^ considered social and environmental factors, and used buffer and landscape index analysis to analyze the changes in the landscape pattern of the Rio Doce State Park in Brazil. They believed that the substantial reduction of farmland area should be given enough attention. Different from this reference, this study established a landscape ecological risk model and studied the aggregation effect of ecological risks through autocorrelation analysis. In addition, although some literatures do not establish ecological risk models, they take other factors such as social and economic into consideration. Through regression analysis, they studied the relationship between landscape pattern and socio-economic factors qualitatively or quantitatively, which had a certain positive effect on the development of ecological economy^63,64^, and is also some of the shortcomings of this study. By synthesizing social, historical, economic, environmental and other factors, constructing an ecological risk assessment model under the influence of multiple factors, thus laying the foundation for the establishment of a scientific and practical ecological development model, may be a valuable future research direction.

Changes in the landscape pattern of land use will inevitably lead to changes in regional ecological functions. Therefore, studying ecological risk changes from the perspective of its landscape structure helps to objectively reflect the ecological risk pattern of highways (and probably other linear infrastructures as well).

## Conclusions

Combining the information of land use and land cover data with a Chinese highway vector data, this research study has evaluated the impact of highway construction on the landscape. As a result, an ecological risk model for a high-altitude plateau area has been proposed using a geographic information system combined with statistical analysis. The following conclusions are obtained from the Qumei to Gangba highway section (Tibet autonomous region), where this model has been implemented:

1. From 2016 to 2020, the major land use type in the buffer zone along the highway was grassland, accounting for more than 52% of the surrounding area. This land use was followed by woodlands and unused lands. The overall trend of land use change was a decrease in grassland area. Conversely, the area of woodland, cultivated land, construction land and water-based land increased. The type of transfer mainly involved the conversion of grassland to woodland, and a balanced mutual conversion between grassland and unused land. This means that before the highway construction, the area of grassland and unused land transferred to each other was almost the same. However, after highway construction, the area of grassland converted to unused land was comparatively much larger, whereas he mutual transfer area of other land use types was negligible.
2. The average values of the ecological risk indices at 2016, 2018 and 2020 were 0.2316, 0.2217 and 0.2822. This indicates that the ecological risk intensity of the study area also showed different trends before and after the highway construction. Before the highway construction, the ecological risk slightly reduced. Whereas after the road construction, the ecological risk increased significantly.
3. During the study period, the transfer of ecological risks mainly involved transfers between adjacent risk levels (cross-risk transfers seldom occurred). However, before the highway construction, the ecological risks were mainly transferred to the lower-level risk level. After the construction project, the ecological risks were mainly transferred to a higher risk level.
4. Global autocorrelation analysis performed with the Moran index indicated that the ecological risk index along the highway had a high degree of positive correlation with the spatial distribution. In other words, adjacent land uses interacted with each other and showed a high degree of spatial similarity. In terms of time series, the spatial agglomeration of land plots with similar ecological risk levels did not show an obvious trend. Based on the local autocorrelation analysis, though, it was found that the spatial distribution of ecological risk index was dominated by high–high aggregation and low–low aggregation. The areas of high-high aggregation increased significantly during the highway construction period and this evidences that the highway construction had an important influence on the spatial distribution of ecological risks.

This study has qualitatively and quantitatively analyzed the process and ecological effects of highway construction on the ecological environment from the perspective of the landscape pattern. It has also provided some new approaches for the study of infrastructure-related ecological risks in high-altitude plateaus. The study has mainly considered the impact assessment of a single highway, but have neglected other roads along the route. Similarly, the corresponding relationship between different buffer distances and landscape ecological risk has not been discussed. Those simplifications will be analysed in further research work, but we find it is highly unlikely that they could have affected the major conclusions from this study.

Due to the fragility of the plateau landscape in Tibet and the complexity of the geological conditions, how to carry out a quantitative analysis of the impact of construction roads on the ecological environment for this type of area has become the key to ecological environmental assessment. This study constructed a plateau high-altitude landscape ecological risk assessment model and analyzed the impact of highway construction on ecological risks dynamically. Its research results provided technical support for the construction of differentiated ecological restoration programs, and also provided a new research perspective and research foundation for ecological risk assessment of engineering construction projects in high-altitude plateau.

## References

[1] Tsering, D. Transport development in Tibet since the democratic reform in 1959. *J*. *Tibetan Stud.* 76–85 (2019).

[2] Yan X (2013). Relationships between heavy metal concentrations in roadside topsoil and distance to road edge based on field observations in the Qinghai-Tibet Plateau, China. Int. J. Environ. Res. Public Health..

[3] Berling-Wolff S, Jianguo WU (2004). Modeling urban landscape dynamics: A case study in Phoenix, USA. J. Urban Ecosyst..

[4] Jianzhou G, Yansui L, Beicheng X (2009). Spatial heterogeneity of urban land-cover landscape in Guangzhou from 1990 to 2005. J. Geogr. Sci..

[5] Pan L, Zhang H, Liu A (2015). Analysis of threshold of road networks effecting landscape fragmentation in Chongqing. J. Ecol. Sci..

[6] Paukert CP, Pitts KL, Whittier JB, Oldenc JD (2011). Development and assessment of a landscape-scale ecological threat index for the Lower Colorado River Basin. J. Ecol. Indic..

[7] Li, H., Yu, Q., Li, N., Wang, J. & Yang, Y. Study on landscape dynamics and driving mechanisms of the Shudu Lake catchment wetlands in Northwest Yunnan. *J*.* West China Forest. Sci.* **42**(3), 34–39 (2013).

[8] Yunqing, H., Jinxi, W. & Hong, J. The dynamics of land cover change pattern and landscape fragmentation in Jiuzhaigou Nature Reserve, China. *J*. *Proc. SPIE Int. Soc. Opt. Eng.* **7498**, 74983P (2009).

[9] Hu, L. *et al.* Landscape pattern in Nanwenghe nature reserve and its driving forces. *J*. *Protect. Forest Sci. Technol.* **0**(7), 18–21 (2015).

[10] Li, X. *et al.* Land use/cover and landscape pattern changes in Manas River Basin based on remote sensing. *J*. *Int. J. Agric. Biol. Eng.* **13**(5), 141–152 (2020).

[11] Andrejs, R. & Merkurjevs, J. Software tool implementing the fuzzy AHP method in ecological risk assessment. *J*. *Inform. Technol. Manag. Sci.* **20**(1), 34–39 (2017).

[12] Peng J, Dang W, Liu Y, Zong M, Hu X (2015). Research progress and prospect of landscape ecological risk assessment. J. Acta Geogr. Sin..

[13] Xu Y, Fu B, Lü H (2010). Research on landscape pattern and ecological processes based on landscape models. J. Acta Ecol. Sin..

[14] Forman R (1998). Road ecology: A solution for the giant embracing us. J. Landsc. Ecol..

[15] Minxi W, Shiliang L, Baoshan C, Min Y (2008). Impacts of hydroelectric project construction on nature reserve and assessment. J. Acta Ecol. Sin..

[16] Yang K, Deng X, Xue-Ling LI, Wen P (2011). Impacts of hydroelectric cascade exploitation on river ecosystem and landscape: A review. J. Acta Ecol. Sin..

[17] Chen LD, Wang JP, Jiang CL, Zhang HP (2010). Quantitative study on effect of linear project construction on landscape pattern along pipeline. J. Sci. Geogr. Sin..

[18] Qianqian H, Luomeng C, Shanlin W (2009). Impact of expressway on land use and landscape pattern: A case study of Putan Guai to Chenghao section of Inner Mongolia Provincial Highway 103. J. Environ. Protect. Sci..

[19] Huang Y, Li Y, Ying H (2015). Responses of Chongqing-Yi Expressway to land use change and landscape pattern. J. Nat. Resourc..

[20] Keken, Z., Sebkova, M. & Skalos, J. Analyzing land cover change—The impact of the motorway construction and their operation on landscape structure. *J*. *Geogr. Inform. Syst.* **6**(5), 559–571 (2014).

[21] Mengna H, Ting M (2019). Assessing the impacts of China's road network on landscape fragmentation and protected areas. J. Geo-inform. Sci..

[22] Mothorpe C, Hanson A, Schnier K (2013). The impact of interstate highways on land use conversion. J. Ann. Reg. Sci..

[23] Wu C-F, Lin Y-P, Chiang L-C, Huang T (2014). Assessing highway's impacts on landscape patterns and ecosystem services: a case study in Puli Township, Taiwan. J. Landsc. Urban Plan..

[24] Jia L, Lei T, Yan SH (2011). Environmental impact analysis and control measures in tunnel construction. J. Appl. Mech. Mater..

[25] Wang M (2015). Analysis of high-speed railway construction on ecological environment impact and environmental protection contribution. J. Railway Constr. Technol..

[26] He Y, Xiong C (2011). Environmental impact of waste slurry in pile foundation construction of high-speed railway bridges and its countermeasures. J. Adv. Mater. Res..

[27] Jing C (2020). Influence of cross-sea bridge project on water quality and ecological environment of nearby sea and its tracking, monitoring and verification. J. Ocean Dev. Manag..

[28] Jianhua X, Mingquan W, Shijian Z, Zheng N (2020). Remote sensing monitoring of ecological and economic impacts of major Railway construction along the Belt and Road. J. Sci. Technol. Eng..

[29] Fang L (2012). On ecological environment impact assessment of metal mine construction project. J. Nonferrous Metals (Min. Sect.)..

[30] Bian B, Lin C, Wu HS (2015). Contamination and risk assessment of metals in road-deposited sediments in a medium-sized city of China. J. Ecotoxicol. Environ. Saf..

[31] Limin Y, Yanhai Z, Rongzu Q, Xisheng H (2015). The influence of regional road construction on landscape ecology on both sides: A case study of Jiangle County, Fujian Province. J. Sichuan Agric. Univ..

[32] Liang Z, Nianlai C (2019). Analysis on the impact of Jinwu Expressway on ecological environment based on comprehensive index evaluation method. J. Environ. Sustain. Dev..

[33] Ting W, Zongmin W (2015). Study on eco-environmental impact assessment system of highway construction. J. Resourc. Econom. Environ. Protect..

[34] Igondova E, Pavlickova K, Majzlan O (2016). The ecological impact assessment of a proposed road development (the Slovak approach). J. Environ. Impact Assessm. Rev..

[35] Chen L, Fu B, Zhao W (2008). Source-sink landscape theory and its ecological significance. J. Front. Biol. China.

[36] Wu J (2013). Spatial differentiation of landscape ecological risk in opencast mining area. J. Acta Ecol. Sin..

[37] Wang J, Cui B, Liu J, Yao H, Juan H (2008). The effect of land use and its change on ecological risk in the Lancang River watershed of Yunnan Province at the landscape scale. J. Acta Sci. Circumstan..

[38] Xie H (2008). Regional eco-risk analysis based on landscape structure and spatial statistics. J. Acta Ecol. Sin..

[39] Jinggang LI, Chunyang HE, Xiaobing LI (2008). Landscape ecological risk assessment of natural/semi-natural landscapes in fast urbanization regions——A case study in Beijing, China. J. Nat. Resourc..

[40] Jie W, Wanqi B, Guoxing T (2020). Temporal and spatial characteristics of landscape ecological risk in Qinghai-Tibet Plateau. J. Resour. Sci..

[41] Xuegong X, Huiping L, Zaiyi F, Rencang B (2001). Ecological risk assessment of wetland area in Yellow River Delta. J. Acta Sci. Nat. Univ. Pekinensis.

[42] Malekmohammadi B, Blouchi L (2014). Ecological risk assessment of wetland ecosystems using multi criteria decision making and geographic information system. J. Ecol. Indic..

[43] Campos P, Paz T, Lenz L, Qiu Y, Paz I (2020). Multi-criteria decision method for sustainable watercourse management in urban areas. J. Sustain..

[44] Peng L (2019). Research on ecological risk assessment in land use model of Shengjin Lake in Anhui province, China. J. Environ. Geochem. Health.

[45] Zhang D, Yang S, Wang Z, Yang C, Chen Y (2020). Assessment of ecological environment impact in highway construction activities with improved group AHP-FCE approach in China. J. Environ. Monit. Assess..

[46] Luan B (2019). Evaluating green stormwater infrastructure strategies efficiencies in a rapidly urbanizing catchment using SWMM-based topsis. J. Clean. Prod..

[47] Ramya, S. & Devadas, V. Integration of GIS, AHP and TOPSIS in evaluating suitable locations for industrial development: A case of Tehri Garhwal district, Uttarakhand, India. *J*. *Clean. Prod.* **238**, 117872 (2019).

[48] Koc, K., Ekmekciolu, M. & Zger, M. An integrated framework for the comprehensive evaluation of low impact development strategies. *J*.* Environ. Manag.* **294**, 113023 (2021). 10.1016/j.jenvman.2021.113023 34119982

[49] Xiumei T, Yu L, Yanmin R, Yuchun P, Xingyao H (2016). Study on change of land use and ecosystem service value along expressway. J. China Agric. Univ..

[50] Fei Z, Shanjiang Y, Dongfang W (2018). Ecological risk assessment due to land use/cover changes (LUCC) in Jinghe County, Xinjiang, China from 1990 to 2014 based on landscape patterns and spatial statistics. J. Environ. Earth Sci..

[51] Rangel-Buitrago N, Neal WJ, de Jonge VN (2020). Risk assessment as tool for coastal erosion management. J. Ocean Coast. Manag..

[52] Mo W, Wang Y, Zhang Y, Zhuang D (2017). Impacts of road network expansion on landscape ecological risk in a megacity, China: A case study of Beijing. J. Sci. Total Environ..

[53] Getis A, Ord JK (2010). The analysis of spatial association by use of distance statistics. J. Springer Berlin Heidelberg..

[54] Yingxue Z, Wenbo M, Yong W, Dafang Z (2017). Impact of land use change on landscape pattern around expressways in Beijing. J. Geo-Inform. Sci..

[55] Gang Z, HuiJun G, Guang Z (2014). Changes of wetland landscape pattern in arid inland area of Northwest China: A case study of inner flow area in Junggar, Xinjiang. J. Arid Land Resourc. Environ..

[56] Haihang W, Qianhui Z, Jiayao Z, Chunguo Z (2018). Analysis on dynamic change of landscape pattern of land use in Zhushan County. J. Forest Resourc. Manag..

[57] Shiliang L, Zhifeng Y, Baoshan C, Shu G (2005). Impact of road on landscape and ecological risk assessment: A case study of Lancang River Basin. J. Chin. J. Ecol..

[58] Yuan, Y. *et al.* Flood-landscape ecological risk assessment under the background of urbanization. *J*. *Water.* **11**(7), 1418 (2019).

[59] Xie H, Wang P, Huang H (2013). Ecological risk assessment of land use change in the Poyang lake Eco-economic zone, China. J. Int. J. Environ. Res. Public Health..

[60] Fengjiao X, Xiao L (2018). Ecological risk pattern in coastal areas of Jiangsu Province based on land use change. J. Acta Ecol. Sin..

[61] Mann D, Anees MM, Rankavat S, Joshi PK (2020). Spatio-temporal variations in landscape ecological risk related to road network in the Central Himalaya. J. Hum. Ecol. Risk Assess..

[62] Oliveira BRD, Costa ELD, Carvalho-Ribeiro SM, Maia-Barbosa PM (2020). Land use dynamics and future scenarios of the Rio Doce State Park buffer zone, Minas Gerais, Brazil. J. Environ. Monit. Assessm..

[63] Li, Y., Sun, Y. & Li, J. Heterogeneous effects of climate change and human activities on annual landscape change in coastal cities of mainland China. *J*. *Ecol. Indic*. 10.1016/j.ecolind.2021.107561 (2021).

[64] Dadashpoor H, Azizi P, Moghadasi M (2019). Land use change, urbanization, and change in landscape pattern in a metropolitan area. J. Sci. Total Environ..

